# In Vitro Inhibition of Influenza Virus Using CRISPR/Cas13a in Chicken Cells

**DOI:** 10.3390/mps4020040

**Published:** 2021-06-08

**Authors:** Arjun Challagulla, Karel A. Schat, Timothy J. Doran

**Affiliations:** 1 CSIRO Health and Biosecurity, Australian Centre for Disease Preparedness, Geelong 3220, Australia; arjun.challagulla@csiro.au; 2 Department of Microbiology and Immunology, College of Veterinary Medicine, Cornell University, Ithaca, NY 14853, USA; kas24@cornell.edu

**Keywords:** chicken, CRISPR/Cas13a, crRNA, influenza A virus (IAV)

## Abstract

Advances in the field of CRISPR/Cas systems are expanding our ability to modulate cellular genomes and transcriptomes precisely and efficiently. Here, we assessed the Cas13a-mediated targeted disruption of RNA in chicken fibroblast DF1 cells. First, we developed a Tol2 transposon vector carrying the Cas13a-msGFP-NLS (pT-Cas13a) transgene, followed by a stable insertion of the Cas13a transgene into the genome of DF1 cells to generate stable DF1-Cas13a cells. To assess the Cas13a-mediated functional knockdown, DF1-Cas13a cells were transfected with the combination of a plasmid encoding DsRed coding sequence (pDsRed) and DsRed-specific crRNA (crRNA-DsRed) or non-specific crRNA (crRNA-NS). Fluorescence-activated cell sorting (FACS) and a microscopy analysis showed reduced levels of DsRed expression in cells transfected with crRNA-DsRed but not in crRNA-NS, confirming a sequence-specific Cas13a mediated mRNA knockdown. Next, we designed four crRNAs (crRNA-IAV) against the PB1, NP and M genes of influenza A virus (IAV) and cloned in tandem to express from a single vector. DF1-Cas13a cells were transfected with plasmids encoding the crRNA-IAV or crRNA-NS, followed by infection with WSN or PR8 IAV. DF1 cells transfected with crRNA-IAV showed reduced levels of viral titers compared to cells transfected with crRNA-NS. These results demonstrate the potential of Cas13a as an antiviral strategy against highly pathogenic strains of IAV in chickens.

## 1. Introduction

Influenza A virus (IAV), belonging to the family of *Orthomyxoviridae*, is a negative sense single-stranded RNA (ssRNA) virus that causes severe disease in a wide variety of avian and mammalian species. The genome of IAV consists of eight viral RNA (vRNA) segments that encode at least 11 proteins. Because of the tendency of IAV to acquire mutations, new strains emerge frequently, requiring new vaccines and/or the development of novel antiviral strategies [1]. Previous studies using RNA interference (RNAi)-mediated targeting of essential viral genes showed a potent inhibition of IAV replication in cells and embryonated chicken eggs [2,3]. However, issues such as off-targeting, delivery and cellular toxicity have complicated the potential of RNAi for the treatment of viral diseases [4]. The recent advances in CRISPR/Cas-based genome engineering tools provide an opportunity to develop novel disease mitigation strategies for the control of IAV in the chicken.

The CRISPR/Cas system is an adaptive immune mechanism protecting bacteria and archaea from invading RNA and DNA viruses [5]. The CRISPR/Cas system is broadly classified into class 1 and class 2 and sub-classified into six types (type I to VI), based on the protein constituents of Cas effector complexes. Class 2 CRISPR effectors, e.g., Cas9 and Cas12, type II and V, respectively, are in widespread use to induce targeted sequence-specific mutations into cellular DNA. In contrast, Cas13, a class 2 type VI effector protein, mediates sequence-specific RNA-directed ssRNA recognition and cleavage [6].

Abudayyeh and colleagues (2017) demonstrated that Cas13a from *Leptotrichia wadei* (LwaCas13a) directly targets RNA and mediates robust messenger (mRNA) knockdown by expressing single or multiple crRNA and Cas13a in eukaryotic cells [7]. The CRISPR/Cas13a system consists of the Cas13a protein and approximately 60 nucleotides (nt) CRISPR RNA (crRNA), which encode a customizable 22–28 nt protospacer sequence that guides the Cas13a protein to a targeted degradation of mRNA. Targeted ssRNA cleavage is mediated by the higher eukaryotic and prokaryotic nucleotide-binding (HEPN) domain of Cas13a. Currently, there are four Cas13 subtypes (Cas13a–Cas13d) that have been harnessed for transcript targeting or editing in a range of eukaryotic systems and to abrogate the replication of RNA viruses [7,8,9]. However, the Cas13a-mediated knockdown of RNA in chicken cells has not yet been reported.

In this study, we examined the Cas13a-mediated targeted knockdown of RNA molecules in chicken cells. We designed crRNAs against two exogenous targets (DsRed and IAV) and assessed their functionality in the chicken fibroblast DF1 cell line stably expressing Cas13a. Our results demonstrate that Cas13a can be used for the targeted knockdown of RNA and as a potential antiviral strategy for the control of RNA viruses in chicken cells.

## 2. Materials and Methods

### 2.1. Plasmids

The pC014-LwaCas13a-msGFP (Addgene No. #91902) and pC0040-LwaCas13a crRNA expression vector (plasmid No. Plasmid #103851) were obtained from Addgene (Watertown, MA, USA). The miniTol2 and Tol2 transposase (pTrans) constructs were a gift from Professor S. C. Ekker, Mayo Clinic Cancer Center, Rochester, MN, USA. The DsRed expressing plasmid (pDsRed-Express, Catalogue. No. 632535) was purchased from Clonetech Laboratories (Mountain View, CA, USA).

To generate a Tol2 transposon vector that carries the Cas13a transgene, the Cas13a-msGFP region in the LwaCas13a-msGFP vector was digested with SpeI and BstZ171 and directionally cloned into the previously described Tol2 vector [10] to obtain Tol2-Cas13a-msGFP (referred to as pT-Cas13a).

### 2.2. Design and Cloning of crRNA into the Expression Vector

The crRNAs targeting DsRed or IAV genome (PB1, NP and M) (Table 1) were designed following the minimal requirements specified by Abudayyeh et al., 2017. The crRNAs were 28 bp long, with an A, T or C protospacer flanking sequence (PFS) at the 3′ end. To clone the crRNAs into the pC0040 vector, we followed the previously described protocol [11]. Briefly, complementary oligos encoding the crRNA sequences with CACC or AAAA overhangs were synthesized and inserted into the BbsI restriction enzyme (RE) site of the pC0040 vector. The inserted clones were verified by Sanger sequencing (Micromon Genomics, Australia). The list of oligonucleotide sequences used in this study is provided in Table 2.

To generate a vector carrying four crRNA-IAV expression cassettes in tandem, we first cloned each IAV crRNA individually into pC0040. Next, we PCR-amplified the expression cassette region (hU6 promoter, crRNA, repeat and terminator) with introduced XhoI and SalI RE sites using primers TD1022 (Forward) and TD1023 (Reverse). The resulting PCR products were ligated into a pGEM-T easy vector to generate four individual PGEM-T easy vectors, allowing the assembly of multiple crRNA expression cassettes in tandem, as previously described [12]. Finally, the pGEM-T easy vector carrying four IAV crRNAs was generated, and the orientation of each expression cassette was verified by Sanger sequencing. The crRNAs vectors targeting DsRed, IAV or non-specific sequences are referred to as pcrRNA-DsRed, pcrRNA-IAV or pcrRNA-NS, respectively.

### 2.3. Cell Culture and Transfections

DF1 cells were maintained as previously described [13]. To generate the DF1-Cas13a cell line, DF1 cells were seeded at a density of 2.5 × 10^6^ per well in a 6-well plate 24 h prior to transfection. The transfection mix consisted of 10 µL of Lipofectamine 2000 (L2000) (Catalogue No. 11668019, Thermo Fisher Scientific, Waltham, MA, USA) complexed with 2 μg of pT-Cas13a plasmid and pTrans or irrelevant plasmid following the manufacturer’s instructions. Stably transfected cells were sorted based on GFP expression at 14 days post-transfection and expanded in culture. Additional rounds of sorting were performed to improve the purity of the DF1-Cas13a cells.

To test knockdown of DsRed mRNA, DF1-Cas13a cells were seeded at a density of 2 × 10^4^ cells per well and allowed to grow to 90% confluency overnight. The cells were transfected with 900 ng of pcrRNA-DsRed or pcrRNA-NS and 100 ng of pDsRed-Express, complexed with 3 μL of a L2000 reagent as per the manufacturer’s instructions.

### 2.4. DsRed Knockdown Assays

DsRed expression was examined at 48 h post-transfection using fluorescence microscopy (Leica DMLB). To perform the FACS analysis, transfected DF1 cells were harvested and resuspended in a FACS buffer (5% FCS in PBSA). The cells were analyzed using a BD FACSAria II (BD Biosciences, BD Biosciences, San Jose, CA, USA) equipped with 530 nm and 561 nm lasers with 530/30 (GFP) and 582/15 (DsRed) emission filters. From each sample, approximately 30,000–50,000 cells were gated for analysis using standard forward/side scatter (FSC/SSC) parameters. To assess the DsRed knockdown, the mean fluorescence intensity (MFI) values were normalized as a percentage of the negative control (pcrRNA-NS), from two independent transfection experiments.

### 2.5. IAV Infection

DF1-Cas13a cells were seeded at a density of 2 × 10^4^ cells per well in 24-well plates. After 24 h, the cells were transfected with 1.5 μg of pcrRNA-IAV or pcrRNA-NS vectors complexed with 3 µL of L2000. The transfected cells were maintained in a 0.25% FCS maintenance medium. To maximize the transfection rates, an additional round of transfection was performed after the 1st transfection. Drained monolayers of transfected cells were infected after 24 h with 100 μL of A/WSN/1933(H1N1) (WSN) or A/Puerto Rico/8/1934 (H1N1) (PR8), which was equivalent to an MOI of 0.1. Following the adsorption for 1 h, the cells were maintained in 0.25% FCS maintenance media for cells infected with WSN or maintenance media with 2 μg/mL TPCK-trypsin (Sigma-Aldrich) for the cells infected with PR8. Supernatant fluids were collected at 24 h post infection (hpi) to determine the viral titers using plaque assays.

### 2.6. Plaque Assay

To determine if Cas13a specifically reduced virus replication, plaque assays were performed using MDCK cells as previously described [14]. Briefly, drained MDCK monolayers were inoculated with 400 μL of 10-fold serial dilutions of viral supernatant fluids. After adsorption for 1 h, the monolayers were overlaid with 3 mL of media containing 1% agar and incubated at 37 °C with 5% CO_2_ until the plaques were visible at three days post inoculation. The plaques were visualized by staining with crystal violet and enumerated, and the PFU of the supernatant fluids was calculated.

### 2.7. Statistical Analysis

All statistical analyses were carried out using the GraphPad Prism 7 software. To determine the significance in the reduction of virus replication in DF1-Cas13a cells transfected with crRNA-NS or crRNA-AIV, we performed two-tailed, unpaired *t*-tests with the Mann–Whitney test. *p*-values less than 0.05 were considered significant.

## 3. Results

**Figure 1:**
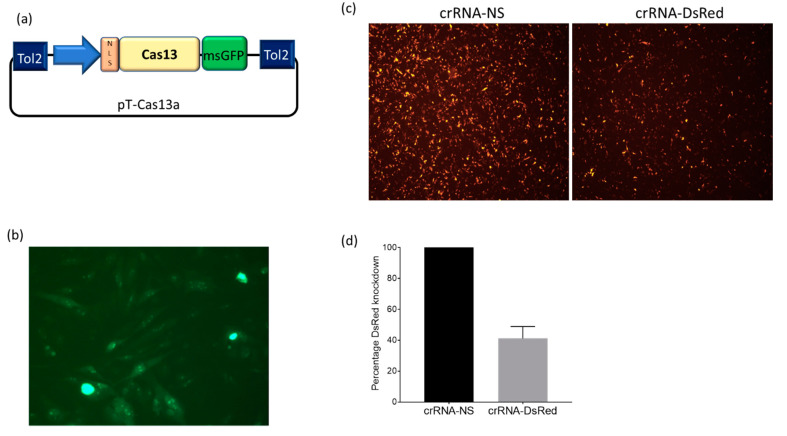
Evaluation of DsRed knockdown by Cas13a in stable DF1-Cas13a cells. (**a**) Schematic of pT-Cas13a vector carrying the Cas13-msGFP-NLS transgene insert sequence; (**b**) Fluorescence microscopy image of DF1-Cas13a cells transfected with pT-Cas13a and pTrans vectors. Stable GFP expressing cells were sorted using flow cytometry at 2 weeks post-transfection to generate a stable DF1-Cas13a cell line; (**c**) Fluorescence microscopy images of DF1-Cas13a cells transfected with 100 ng pDsRed and 900 ng of crRNA-DsRed or crRNA-NS. Images taken at 48 h post-transfection; (**d**) Flow cytometry analysis of DsRed knockdown in DF1-Cas13a cells transfected with DsRed-crRNA or NS-crRNA 48 h post-transfection. The MFI values were normalized as a percentage of the negative control crRNA-NS (100%). Each value is the mean with standard deviation (2 replicates). Results are representative of 2 separate experiments (Figure S2).

To test whether Cas13a could mediate the targeted degradation of mRNA in chicken cells, we began by modifying our previously published miniTol2 transposon vector (Tol2) to carry a Cas13a-msGFP transgene to generate pT-Cas13a (Figure 1a). To generate a DF1 cell line stably expressing Cas13a, we co-transfected the pT-Cas13a and pTrans constructs into DF1 cells and maintained the cells in culture for 2 weeks. Stable DF1-Cas13a cells were sorted based on stable GFP expression using FACS (Figure 1b). To examine if the stably integrated Cas13a transgene is functional and able to mediate targeted mRNA degradation, we selected DsRed mRNA as our target, expressed from pDsRed-Express. DF1-Cas13a cells were co-transfected with plasmids encoding pDsRed and pcrRNA-DsRed or pcrRNA-NS, in two separate wells per treatment group. The functional knockdown effect of the Cas13a and crRNA-DsRed complex on DsRed expression was measured at 48 h post-transfection. Fluorescent microscopy showed that the monolayers transfected with crRNA-DsRed had less DsRed fluorescence than the monolayers transfected with NS-crRNA (Figure 1c). To quantify the reduction of DsRed fluorescence, a FACS analysis was performed (Figure S1). We observed a ~60% reduction in mean fluorescence intensity (MFI) in cells transfected with pcrRNA-DsRed (average MFI: 6679) compared to pcrRNA-NS (average MFI: 11,448), as demonstrated in two independent replicates per treatment group (Figure 1d).

**Figure 2:**
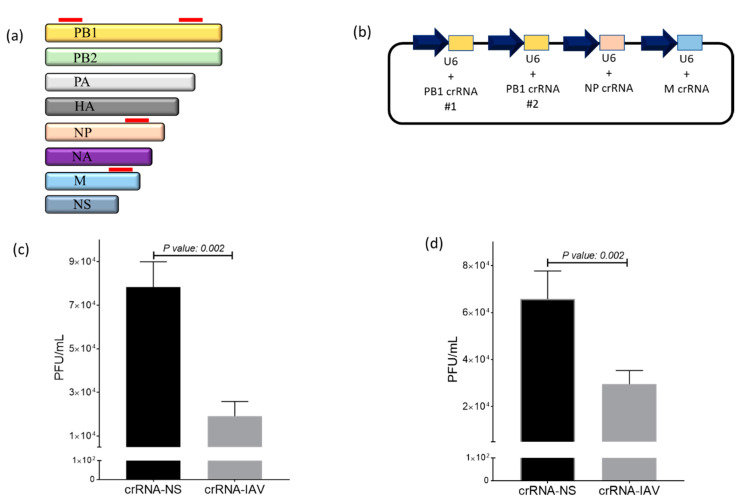
Evaluation of IAV inhibition using CRISPR/Cas13 in DF1 cells. (**a**) Schematic diagram of designed crRNAs in the IAV viral genome. A redline on the PB1, NP and M gene denotes the position of each crRNA; (**b**) Schematic of the pcrRNA-IAV construct developed to express four crRNAs targeting the PB1, NP and M genes of IAV; (**c**,**d**) DF1-Cas13a cells were transfected with pcrRNA-IAV or pcrRNA-NS 24 h and infected with WSN (**c**) and PR8 (**d**) at an MOI of 0.1. Supernatant fluids were collected at 24 hpi to measure viral replication by plaque assays. The results are the pool of two separate experiments (*n* = 6). The error bars correspond to standard deviation. The *p*-value indicates significance between the two treatment groups.

Following the confirmation of the Cas13a-mediated sequence-specific targeting of DsRed mRNA, we next evaluated whether Cas13a can be programmed to target IAV in DF1 cells. Because Cas13a can recruit multiple crRNAs and mediate the sequence-specific targeting of corresponding target mRNAs, we chose a combinatorial targeting approach by simultaneously expressing crRNAs targeting the PB, NP and M genes of the influenza virus (Figure 2a). We constructed a crRNA expression vector encoding four anti-influenza crRNAs (pIAV-crRNAs) that were each cloned using the human U6 promoter to drive the expression (Figure 2b). For virus inhibition studies, we selected two low pathogenic H1N1 influenza strains (WSN and PR8) that efficiently replicate in chicken DF1 cells. To examine an anti-viral effect, DF1-Cas13a cells were transfected with pIAV-crRNA or pNS-crRNA and infected with WSN or PR8 at a MOI of 0.1. Each experiment was conducted twice and consisted of three independent replicates per treatment group. To accurately quantify the reduction of viral replication, supernatant fluids were collected after 24 h, and viral titers were measured by plaque assay. For WSN infection, supernatant fluids from DF1-Cas13a cells transfected with pIAV-crRNAs (average titer: 19,166 ± 6645.8 pfu/mL, *n* = 6) had an approximately four-fold reduction of viral titers compared to pNS-crRNAs transfected cells (average titer: 78,333 ± 11,690.4 pfu/mL, *n* = 6) (*p*-value = 0.002) (Figure 2c). For PR8, a two-fold reduction was observed in cells transfected with pcrRNA-IAV (average titer: 29,583 ± 5791.5 pfu/mL, *n* = 6) relative to pcrRNA-NS (average titer: 65,833 ± 11902.3 pfu/mL, *n* = 6) (*p*-value = 0.002) (Figure 2d). These results demonstrate that Cas13a+crRNA-IAV showed a sequence-specific inhibition of IAV.

## 4. Discussion

We demonstrated the Cas13a-mediated sequence-specific targeting of RNA in chicken cells by the transient expression of crRNAs in a DF1 cell line stably expressing Cas13a. Because Cas13a expression plasmids are large and could potentially effect transfection efficiency, we generated a stable DF1-Cas13a cell line which was used for transient transfection with pcrRNAs. The knockdown of DsRed fluorescence in cells transfected with pcrRNA-DsRed but not with pcrRNA-NS confirms that the stable expression of Cas13a is tolerated and induces the sequence-specific knockdown of DsRed in a sequence-specific manner in chicken cells. These data suggest that Cas13a can be used for the knockdown of endogenous mRNA for functional studies, a potential alternative to existing siRNA knockdown approaches. One of the apparent benefits of using Cas13a, instead of siRNAs, is its high degree of precision while enabling robust mRNA degradation [7]. Furthermore, the RNA sequencing analysis of Cas13a+crRNA-treated cells did not induce any off-target effects at the transcriptomic level [7]. As crRNAs are transiently supplied into DF1-Cas13a cells, the likelihood of off-targets in our experiments is very low.

IAVs have a complex replication cycle, in which the vRNA segments are imported into the nucleus for transcription and the production of complementary RNA (cRNA), followed by synthesis of new copies of vRNA from the cRNA templates. We used the Cas13a open reading frame linked to the nuclear localization signal (NLS) to facilitate the transportation of the synthesized Cas13a protein from the cytoplasm into the nucleus. Furthermore, the crRNAs were designed to target nascent cRNA viral segments. Indeed, these two design strategies will enable the Cas13a-mediated targeting of IAV in the nucleus instead of the cytoplasm in DF1-Cas13a cells transfected with pcrRNA-IAV. The reduction in titers of WSN and PR8 viruses suggests that IAV replication can be efficiently abrogated by the direct cleavage of cRNA strands induced by the Cas13a-crRNA complex (Figure 2c,d). Previously, a direct comparison of the siRNA-mediated targeting of vRNA and cRNA strands of IAV showed that the cRNA is a likely target for siRNA [2]. Similar studies were performed using Cas13a and found that the targeting of cRNA strands of IAV has efficiently abrogated the replication in human cells [15].

It is worth noting that the combinatorial targeting approach using four crRNAs targeting the PB1, NP and M genes has two advantages. First, the expression of crRNAs from four individual U6 cassettes facilitates elevated crRNA levels in transfected cells, permitting the efficient recruitment of crRNAs by the Cas13a complex for virus interference. A previous study has shown that the simultaneous targeting of the viral genome with multiple shRNAs reduced the risk of escape mutants whilst achieving virus inhibition [16]. Furthermore, the use of multiple crRNAs exhibited a robust Cas13a-mediated inhibition of Porcine reproductive and respiratory syndrome virus compared to a single crRNA [17]. Second, targeting multiple viral segments could potentially reduce the emergence of escape variants and enable a potential coverage, if any resistant mutations are acquired at the target crRNA site. Because the viral polymerase PB1 gene is one of the highly conserved genes of IAV and an essential part of the IAV replication cycle, we targeted PB1 at two sites by designing two crRNAs. Intriguingly, Freije et al. (2019) showed that the Cas13-mediated targeting of IAV in Madin–Darby canine kidney epithelial cells by electroporating plasmids expressing crRNAs and Cas13 did not result in any escape mutants [18]. However, in future studies it would be valuable to investigate if escape mutants were not generated after sequential passages with CRISPR/Cas13a treatment. In conclusion, our combinatorial strategy provided IAV inhibition and could serve as an effective strategy against potential escape mutations.

To our knowledge, this is the first successful report on CRISPR/Cas13a-induced programmable interference of exogenous RNA, including IAV in chicken cells. The data obtained provide the foundation to test the Cas13a antiviral activity against highly pathogenic avian influenza strains such as H5N1 avian influenza or other poultry pathogens. Anti-viral CRISPR/Cas13a transgenes could be effectively inserted into the genome of chickens by methods such as the Tol2 transposon to generate germline transgenic chickens [10,13,19] expressing Cas13a and crRNAs targeting H5N1, followed by the demonstration of an anti-viral effect through in vivo challenge experiments.

## Figures and Tables

**Table 1:** List of crRNAs used in the study.

Target	Sequence (5′-3′)
DsRed	CGACATCCCCGACTACAAGAAGCTGTCC
PB1-#1	TACACCATGGATACTGTCAACAGGACAC
PB2-#2	CTGAGATCATGAAGATCTGTTCCACCAT
NP1	AATGAAGGATCTTATTTCTTCGGAGACA
M	GAACACCGATCTTGAGGTTCTCATGGAA
NS control	ATGCATGCATGCATGCATGCATGCATGC

**Table 2:** List of primers used in the study.

Primer	Sequence (5′-3′)
crRNA-DsRed-F	AAACGGACAGCTTCTTGTAGTCGGGGATGTCG
crRNA-DsRed-R	AAAACGACATCCCCGACTACAAGAAGCTGTCC
crRNA-PB1#1-F	AAACGTGTCCTGTTGACAGTATCCATGGTGTA
crRNA-PB1#1-R	AAAATACACCATGGATACTGTCAACAGGACAC
crRNA-PB1#2-F	AAACATGGTGGAACAGATCTTCATGATCTCAG
crRNA-PB1#2-R	AAAACTGAGATCATGAAGATCTGTTCCACCAT
crRNA-NP-F	AAACTGTCTCCGAAGAAATAAGATCCTTCATT
crRNA-NP-R	AAAAAATGAAGGATCTTATTTCTTCGGAGACA
crRNA-M-F	AAACTTCCATGAGAACCTCAAGATCGGTGTTC
crRNA-M-R	AAAAGAACACCGATCTTGAGGTTCTCATGGAA
crRNA-NS-F	AAACATGCATGCATGCATGCATGCATGCATGC
crRNA-NS-R	AAAAGCATGCATGCATGCATGCATGCATGCAT
TD1022	ACTACCGGTGTCGACCGCCAGAGGGCCTATTTCCCA
TD1023	ACTACCGGTCTCGAGGTGAATTCGAGCTCGGTACC

## Data Availability

Data is contained within the article or Supplementary Materials.

## Supplementary Materials

The following are available online at https://www.mdpi.com/article/10.3390/mps4020040/s1, Figure S1: FACS plots of DsRed knockdown experiment in DF1-Cas13a cells transfected with either crRNA-DsRed or crRNA-NS. Note: The gRNA means crRNA. Figure S2: Fluorescence and bright field images of DsRed knockdown cells.
