# Supplementary material for: In Vitro Inhibition of Influenza Virus Using CRISPR/Cas13a in Chicken Cells
Source: Methods Protoc. 2021 Jun 8;4(2):40. doi: 10.3390/mps4020040 (PMC8293360; doi:10.3390/mps4020040)
Supplement: Supplementary file 1 [file mps-04-00040-s001.zip › mps-1236532-supplementary.pdf]

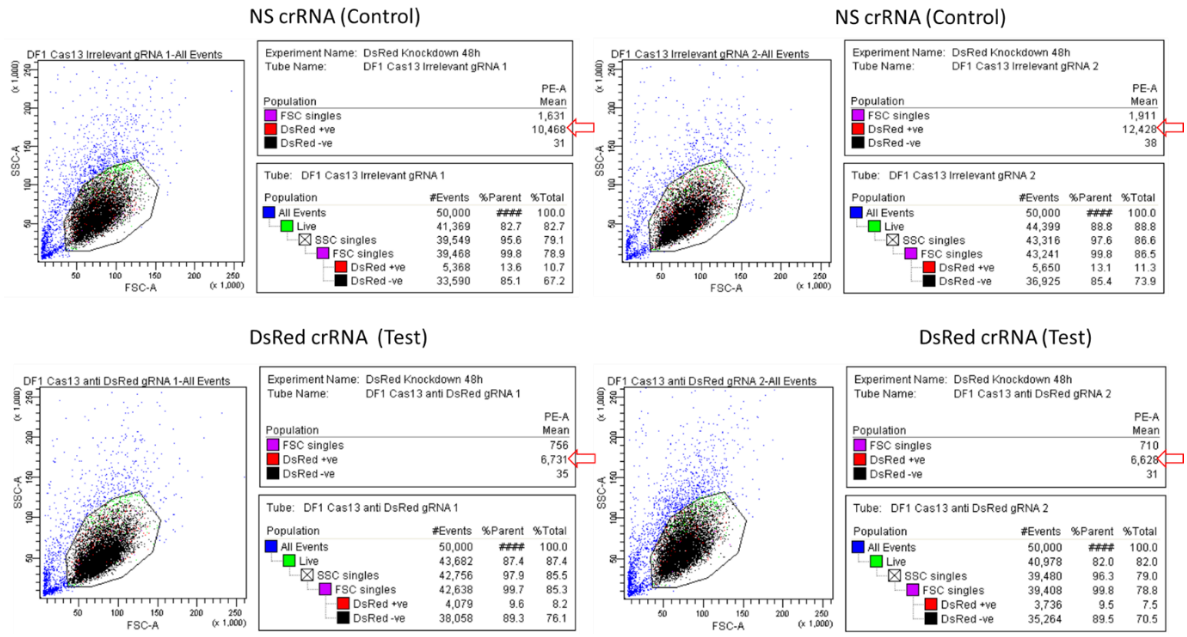

Figure 1. FACS plots of DsRed knockdown experiment in DF1-Cas13a cells transfected with either crRNA-DsRed or crRNA-NS. Note: The gRNA means crRNA.

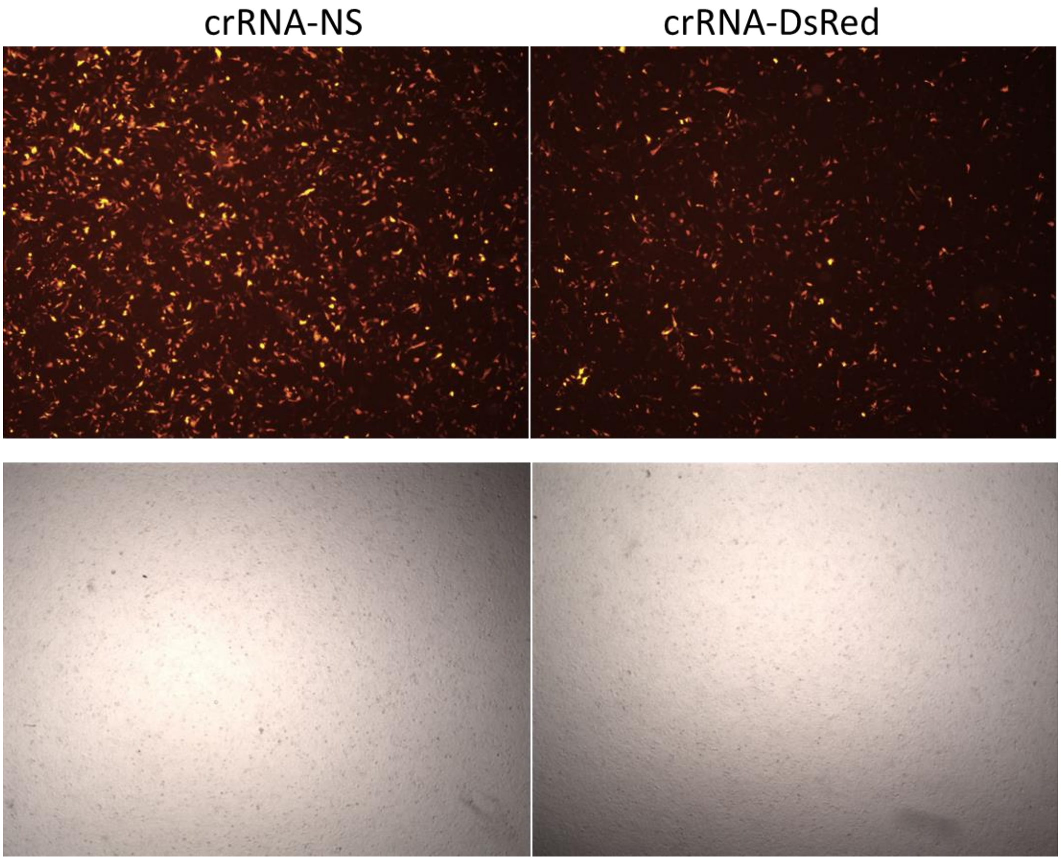

Figure 2. Fluorescence and bright field images of DsRed knockdown cells.
